# Inhibition of *Candida albicans* by Fluvastatin Is Dependent on pH

**DOI:** 10.1155/2009/151424

**Published:** 2009-08-05

**Authors:** Martin Schmidt, Seli Dzogbeta, Michael P. Boyer

**Affiliations:** Department of Biochemistry and Nutrition, Des Moines University, 3200 Grand Avenue, Des Moines, IA 50312, USA

## Abstract

The cholesterol-lowering drug fluvastatin (FS) has an inhibitory effect on the growth of the pathogenic yeast *Candida albicans* that is dependent on the pH of the medium. At the low pH value of the vagina, FS is growth inhibitory at low and at high concentrations, while at intermediate concentrations (1–10 mM), it has no inhibitory effect. Examination of the effect of the common antifungal drug fluconazole in combination with FS demonstrates drug interactions in the low concentration range. Determination of intracellular stress and the activity of the FS target enzyme HMG-CoA reductase confirm our hypothesis that in the intermediate dose range adjustments to the sterol biosynthesis pathway can compensate for the action of FS. We conclude that the pH dependent uptake of FS across yeast membranes might make FS combination therapy an attractive possibility for treatment of vaginal *C. albicans* infections.

## 1. Introduction

Fluvastatin (FS) is a widely prescribed inhibitor of HMG-CoA reductase, the key enzyme in sterol biosynthesis [1]. Inhibition of sterol synthesis by fluvastatin has an antifungal effect on the opportunistic yeast pathogen *Candida albicans * and at high concentrations; the antifungal action of FS is synergistic with the action of other antifungal drugs [2, 3]. However, a combination drug therapy against *C. albicans* in which FS is combined with the commonly prescribed azole drugs seems impractical due to the high concentrations of FS required for an inhibitory effect at physiological pH [2, 3].

FS stands out from the other members of the statin family in that it is a fully synthetic molecule with a terminal carboxylic acid group. Because of its pKa of 5.5, FS is ionized at the physiological pH of 7.4. FS uptake into cells is dependent on pH. The uncharged protonated form of FS in the acidic pH of the intestine is taken up more readily that the charged molecule is present at serum pH [4].

The present study was undertaken to show that FS action on yeast is similarly dependent on the pH of the medium. It is of particular interest to establish if a more effective uptake of FS at low pH leads to synergism of FS with azole drugs at lower readily achievable statin concentrations.

## 2. Materials and Methods

### 2.1. Assessing Yeast Growth

*C. albicans* strains from our collection of clinical isolates [5] were grown in synthetic medium [6] pH-adjusted with 0.1 M Sodium Phosphate buffer. Yeast growth was assayed by a modified microtiter broth dilution method [6]. Stationary cultures of yeast were diluted 1/20 000 in fresh medium, aliquoted to 200 *μ*L with the indicated concentrations of drugs and incubated for 40 hours at 37°C in 96-well microtiter plates. Growth was estimated by recording the optical density at 620 nm (OD620) with a microplate reader. Minimal inhibitory concentrations (MICs) of drugs were determined as doses that reduced OD620 by >50% compared to control [7].

### 2.2. Assessing CDR1 Expression

The expression of the stress-response gene *CDR1* was monitored by recording the fluorescence of *C. albicans* reporter strain CaSA1 in which the expression of green-fluorescent protein is under control of the *CDR1* promoter [8].

### 2.3. Determination of HMG-CoA Reductase Activity

Ten OD600 units of a culture of *C. albicans* strain ATCC10231 in synthetic medium were harvested in mid log phase of growth, washed twice with water and suspended in 300 *μ*L of 100 mM KPO_4_ buffer at pH7.0 supplemented with yeast protease inhibitor cocktail (Sigma-Aldrich, St. Louis, Mo, USA). After addition of 300 *μ*L of acid-washed glass beads, cells were ruptured in a Beadbeater instrument (Biospec, Bartlesville, Okla, USA). After removing cell debris from the glass-bead supernatant, HMG-CoA reductase activity in the crude extract was determined by following the oxidation of NADPH at 340 nm. The assay contained 0.3 mM HMG-CoA, 0.2 mM NADPH, 40 mM Dithiothreitol, and 10 *μ*L of crude lysate in 100 mM KPO_4_ buffer at pH7.0. Activity was normalized to protein concentration in the extract. Averages and standard deviations of 3 independent experiments were calculated [9].

### 2.4. Quantitative PCR

Expression of *UPC2* [10] was assayed by quantitative reverse transcriptase PCR (qPCR). Total RNA was isolated with the Yeast Master Pure RNA kit (Epicentre Biotechnology, Madison, Wis) according to the manufacturer's instructions. qPCR was carried out using the qTaq DNA polymerase kit (Clontech, Mountain View, Calif) at a template concentration of 50 ng/*μ*L. The following primers were used: PMA1 (control 1) 5′ TCCAACCTTTCGATCCTGTC 3′ and 5′ TTCCCAGTGACCTTCACCTC 3′; 16S RNA (control 2) 5′ATGGCCGTTCTTAGTTGGTG 3′ and 5′GCCAAGGGTTATACTCGCTG 3′; UPC2 5′ CAGCACTTTTGGACAAGCAA 3′ and GCTCCACCTGCGTACTCTTC 3′. Averages and standard errors for three independent experiments are given. 

## 3. Results and Discussion

### 3.1. Inhibition of *C. albicans* Growth by FS Is Dependent on Environmental pH

**Figure 1:**
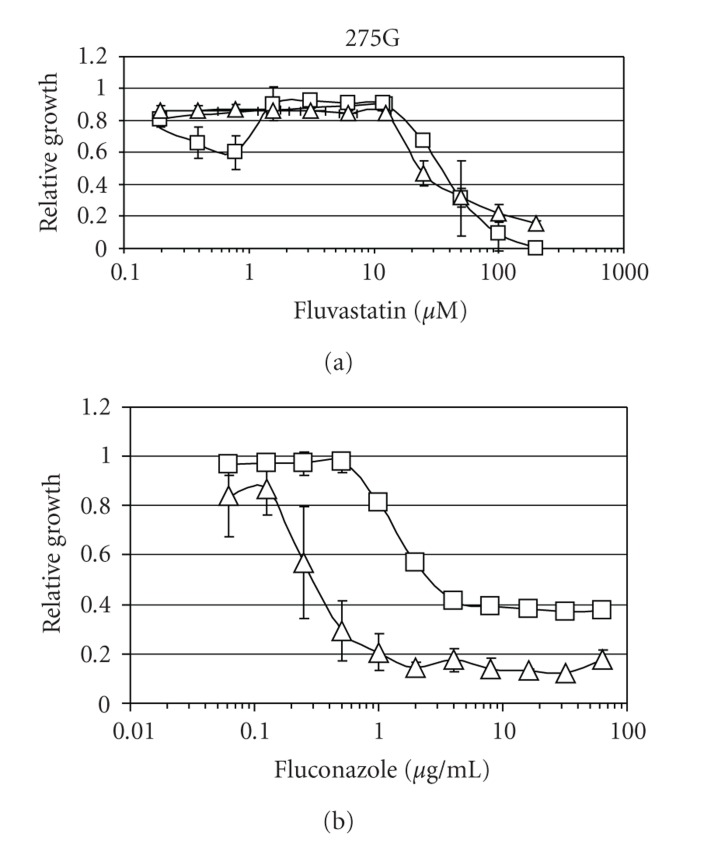
Impact of Fluvastatin (FS) on survival and Fluconazole resistance of *C. albicans*. (a) Growth inhibition of clinical isolate 275G by FS. Squares, pH 4.5; Triangles, pH 7. Relative growth is given as OD620 treated/ OD620 untreated culture at the respective pH (4.5 or 7.0). At pH 4.5, FS causes growth inhibition at low concentrations. (b) FS, at low concentration/low pH, enhances the growth inhibition by fluconazole. Squares: strain 275G grown at pH 4.5 without FZ; triangles: same strain grown with 0.2 *μ*M FS.

The effect of FS on growth of *C. albicans* was examined in four different clinical isolates from our collection. A typical dose-response curve is shown in Figure 1(a). While at a pH of 7.0 FS, it exhibits a simple dose/response correlation, the situation at pH 4.5 is different. Growth inhibition can be observed at low (0.5–1.0 *μ*M) and high (>10 *μ*M) concentrations, but not in the intermediate concentration range of 1–10 *μ*M. Of the 6 statins examined in this study (Atorvastatin, Fluvastatin, Lovastatin, Mevastatin, Pravastatin, Simvastatin), FS was the only drug exhibiting a nonlinear dose response.

The low-dose growth-inhibitory action of FS at low pH values can be understood in light of the previously demonstrated pH dependency of FS uptake across biological membranes [4]. We propose that the protonated uncharged FS molecule has better access to the yeast cytoplasm than the charged molecule and thus can inhibit growth at lower concentrations.

The morphology of statin-treated cells was examined by light microscopy. There was no change in cell shape or size upon exposure to FS (data not shown). 

The low dose/low pH inhibitory effect of FS can be exploited to sensitize *C. albicans* to the action of a common antifungal drug, fluconazole (Figure 1(b)). An azole drug was chosen for the analysis because both azoles and statins inhibit the synthesis of the major fungal membrane lipid ergosterol. While the statins inhibit an early step, the reduction of the steroid precursor hydroxymethylglutaryl-CoA (HMG-CoA), azoles inhibit a later enzymatic step, the demethylation of 14 *α*-lanosterol [11]. 

The fluconazole sensitivity of eight clinical isolates of *C. albicans * was assayed in the presence and absence of 0.5 mM FS. In all strains tested, the addition of FS lowered the MIC for fluconazole by at least a factor of four. 

In order to validate the observed FS-mediated increases and decreases in fluconazole resistance, a checkerboard analysis [3] was performed at pH 4.5. However the low-dose sensitizing effects of FS did not meet the stringent criteria for synergism as put forward by White and coworkers [12].

### 3.2. CDR1-Fluorescence and HMG-CoA Activity Mirror FS Action

**Figure 2:**
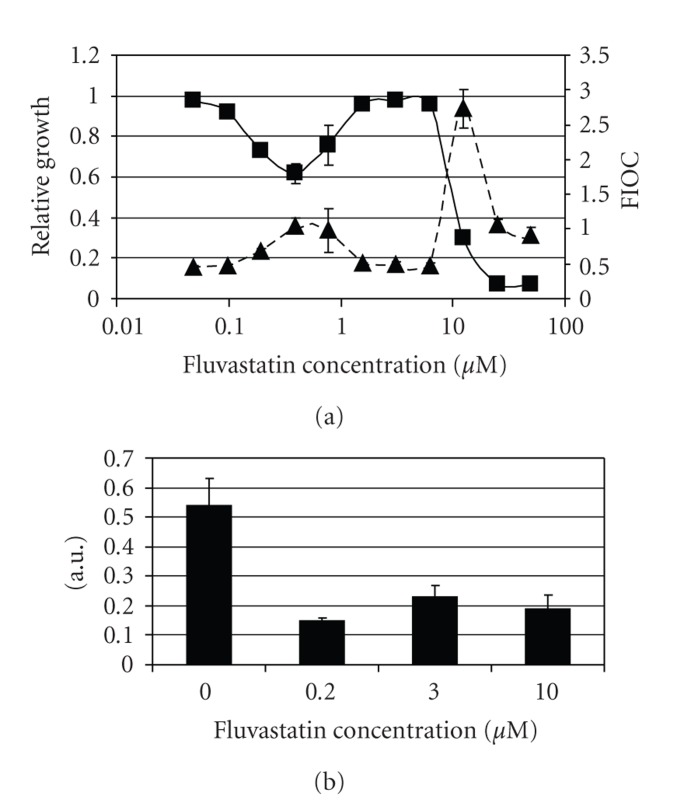
Impact of FS on stress response and HMG-CoA reductase activity in *C. albicans*. (a) Fluorescence and growth of *CDR1 * reporter strain CaSA1 in the presence of FS. Squares: Growth of CaSA1 relative to growth of FS-untreated cells. Triangles: Corresponding GFP fluorescence of samples. The intensity of GFP fluorescence is a measure of *CDR1* expression and thus reflects the strength of the stress response. In the intermediate concentration range of 1–10 *μ*M that does not inhibit growth the cells do not exhibit signs of stress. (b) HMG-CoA reductase activity of strain ATCC10231 in arbitrary units. In the intermediate concentration range, HMG-CoA reductase is not as strongly inhibited as in the low range.

In an effort to better understand the biological implications of the nonlinear FS dose-response curve shown in Figure 1(a), we assessed the intracellular stress levels and HMG-CoA reductase activities of FS-treated *C. albicans*. The expression of the drug exporting pump Cdr1p increases after exposure to a variety of stress factors and is considered a useful reporter for intracellular stress [8]. The FS-induced changes in *CDR1 * gene expression are a mirror image of the drug's growth inhibitory effect (Figure 2(a)). In the intermediate concentration range of 1–10 *μ*M, FS does not induce drug resistance mechanisms. A similar correlation can be observed in the FS-induced changes in HMG-CoA reductase activity. While a low dose of FS inhibits *C. albicans*  HMG-CoA reductase, the intermediate dose inhibits the enzyme significantly less (Figure 2(b)). Induction of the major steroid synthesis regulator *UPC2 *[10], as determined by qPCR, is detectable at low FS concentrations (factor 1.53 +/− 0.28 at 0.5 *μ*M), indicating that FS indeed has a physiological effect in this range. However, *UPC2*  induction does not increase significantly with escalating FS concentrations (factor 1.69 +/− 0.24 at 20 *μ*M) and can thus not explain an adaptive response at intermediate concentrations. 

We propose the following explanation for our observations At a low pH, the uncharged FS molecules enter the cell by diffusion though the membrane. Inside the cell, FS gets trapped by deprotonation at the higher intracellular pH, leading to an intracellular accumulation with subsequent inhibition of ergosterol biosynthesis. If a certain level of inhibition is exceeded, the cell might respond by inducing the activity of the steroid synthesis pathway [10, 13], apparently by a *UPC2*-independent mechanism. This then would lead to adequate production of ergosterol and to a lowering of intracellular stress in the presence of FS. Clearly, further studies are required to fully explain the FS resistance in the intermediate concentration range.

## 4. Conclusions

The data about the pH dependency of FS action on *C. albicans* give insight into the impact of statin therapy on yeast infection. In patients taking FS for the treatment of hypercholesterolemia, the serum concentration of FS falls into the intermediate range of 2–4 *μ*M [14]. No fungistatic or fungicidal action of FS can be expected in this range, as the effective concentration of FS at physiological pH is >12.5 *μ*M. However, at the low pH of the vagina (pH 3.8 to 4.5) low doses of FS are sufficient to reduce *C. albicans* survival. It is an appealing hypothesis that FS can used alone or in combination with azoles to improve fungal clearance from the vagina.
